# First do no harm: Client and staff experiences of negative effects from dialectical behaviour therapy

**DOI:** 10.1111/papt.12578

**Published:** 2025-02-15

**Authors:** Zazie Lawson, Lorna Farquharson

**Affiliations:** ^1^ Department of Psychology and Human Development University of East London London UK

**Keywords:** DBT, dialectical behaviour therapy, iatrogenic harm, negative effects, negative experiences, psychological therapy

## Abstract

**Objectives:**

Research has shown that dialectical behaviour therapy (DBT) is effective in reducing self‐harm and suicidal ideation, but there is also some evidence of negative effects with differences in the reports from clients and staff. However, no research has focused on both groups' understandings of negative effects. This study aimed to explore client and staff experiences of the negative effects from DBT, investigate how their understandings compare, and how staff address any negative effects that arise.

**Methods:**

Eight client participants and seven staff participants, who had experienced or witnessed negative experiences from DBT, engaged in semi‐structured interviews, the transcripts of which were analysed using reflexive thematic analysis.

**Results:**

Four themes relating to client experiences were generated: ‘I'm the problem’, ‘DBT can do no wrong’, ‘No understanding of trauma’ and ‘An unhealthy “blueprint for relationships”’. Five themes relating to staff experiences were generated: ‘It's not me, it's the client’, ‘DBT or nothing’, ‘We don't do ‘why’ in DBT’, ‘We did make some changes’ and ‘Organisational “restrictions”’.

**Conclusions:**

Both staff and clients understood negative effects from DBT to include pathologisation and re‐traumatisation. However, whilst clients related negative effects to the therapeutic relationship, staff highlighted the impact of organisational restrictions. The findings support a number of changes to practice, particularly the need to recognise potential negative effects and provide meaningful informed consent procedures.

## INTRODUCTION

‘Primum non nocere’ or ‘first do no harm’ has become one of the fundamental guiding principles for health care professionals (Travers, 2018). The third principle in the British Psychological Society's (BPS) Code of Ethics and Conduct features a similar notion, stating that psychologists' practice should include ‘the avoidance of harm and the prevention of misuse or abuse of their contributions to society’ (BPS, 2021, p. 18). However, in contrast to the significant interest in the potential negative effects of psychotropic medication (Sharp & Chapman, 2004), until recently there has been very little research into or documentation of the negative effects of psychological therapies (Duggan et al., 2014; Jonsson et al., 2014; Vaughan et al., 2014).

Dialectical behaviour therapy (DBT) is a skills‐based cognitive behavioural therapeutic modality, underpinned by the biosocial model, which is recommended by The National Institute of Health and Clinical Excellence (NICE) for clients who have been given a diagnosis of ‘borderline personality disorder’ (‘BPD’; NICE, 2009). Although there is existing literature regarding both client and staff experiences of DBT, very little of this focuses explicitly on experiences of harm or negative effects.

### Dialectical behaviour therapy

To understand the context in which DBT emerged, it is necessary to take a closer look at the researcher who developed it (Marsha Linehan), and the various influences on her work. Many of the skills taught in DBT programmes today can be traced back to Linehan's personal experiences during a psychiatric hospital admission. For example, after finding that ‘cold pack’ therapy helped to regulate her emotions, Linehan introduced ‘ice diving’ to the DBT skills repertoire. However, other experiences Linehan found less helpful and so attempted to avoid or address. In Linehan's memoir *Building a Life Worth Living* (2020), she reflects on how compassion and attempts to understand her behaviour were in her opinion insufficient. According to Linehan, her own ‘suicidal behaviour’ was being positively reinforced by staff's efforts to help her.

Linehan holds behaviourism in high regard, having completed a post‐doctoral fellowship in behaviour modification. In keeping with this, the origins of DBT are, in essence, behavioural. It emerged from a series of unsuccessful attempts in the 1970s to apply behavioural principles and social learning theory (Staats & Staats, 1963) to the treatment of people referred to as ‘chronically suicidal’ (Linehan & Wilks, 2018). Perhaps unsurprisingly, clients reported that the focus on behavioural change was invalidating and often lead to them withdrawing from therapy, and even in some cases attempting to end their lives (Dimeff & Linehan, 2001). To address these difficulties, Linehan and colleagues introduced several adaptations, including validation and radical acceptance, drawing on Zen and contemplative practices (Linehan & Wilks, 2018). These alterations facilitated a shift from a purely behavioural focus to a more acceptance‐based approach that encouraged temporarily tolerating distressing experiences (Linehan & Wilks, 2018). However, the emphasis on acceptance left some clients feeling hopeless (Linehan, 2020). This balance, or ‘dialectic’, between acceptance and change is the cornerstone of DBT as it is known today.

DBT in its current form purports to serve five key functions: enhancing capabilities, generalising capabilities, increasing motivation, enhancing therapist capabilities and structuring the environment (Lynch et al., 2007). A full DBT programme adopts four modes of treatment: group skills training, individual therapy, telephone coaching and a therapist consultation team (Robins et al., 2010). Skills teaching takes place during weekly two‐hour groups and consists of four modules: mindfulness, interpersonal effectiveness, emotional regulation and distress tolerance (May et al., 2016). All skills teaching has a didactic focus and entails instructions, modelling, coaching and homework assignments (Linehan & Wilks, 2018).

A systematic review of 75 randomised controlled trials (RCTs) found that overall DBT was effective at reducing self‐harm and improving psychological functioning (Storebø et al., 2020). However, the majority of the RCTs conducted had small sample sizes and a high risk of bias (Storebø et al., 2020). Indeed, the efficacy of DBT when compared to alternative psychological treatments remains highly debated (Little et al., 2017).

### Negative effects

Between 40% and 60% of clients do not reach the defined recovery criterion after engaging in psychological therapy (Gyani et al., 2013). In terms of negative effects, prevalence varies both according to therapist and based on client characteristics (Saxon et al., 2017). Indeed, Crawford et al. (2016) found that clients who were from ethnic and sexual minority groups were more likely to report negative effects. However, there is some consistency in the research, which suggests that overall, between approximately five and ten percent of all clients have experiences of negative effects from therapy (Hansen et al., 2002; Hatfield et al., 2010; Lambert, 2013).

Within the research on negative effects from psychological therapy, it has been rare for participants to cite a single cause. Instead, causes have appeared to be multifactorial in nature, consisting of a variety of client, clinician and therapeutic modality factors (Hardy et al., 2017; Jonsson et al., 2016). A model by Curran et al. (2019) outlined the ways in which different factors may interact to contribute to negative effects. The findings from the researchers' synthesis of service user testimony and qualitative research suggested that contextual factors and unmet client expectations contribute to negative therapeutic processes. These processes encompassed therapist behaviours which, in combination with power imbalances, contributed to clients feeling disempowered and silenced. Parry et al. (2016) also helpfully outlined key categories into which possible mechanisms for negative effects may fall, which included the therapeutic relationship, therapist factors, poor fit between client and therapist or intervention, specific intervention risks and organisational factors.

Therapists who rely solely on their clinical judgement are very poor at identifying negative effects in clients (Hannan et al., 2005; Hatfield et al., 2010). In addition, therapists rarely receive specific training on how to identify or respond to negative effects (Bystedt et al., 2014; Castonguay et al., 2010). This, in combination with the findings that it is rare for clients to spontaneously disclose negative effects (Hardy et al., 2017; Horigian et al., 2010), indicate just how important it is to consider formal methods of identifying harm.

Several measures have been published, which have been designed to support the identification and recording of negative effects. The Unwanted Event to Adverse Treatment Reaction checklist (UE‐ATR; Linden, 2013), for example, was developed with the intention of supporting clinicians to identify negative effects in their routine practice. However, it should be noted that the psychometric properties of the UE‐ATR have not yet been researched (Farquharson, 2020). The Experiences of Therapy Questionnaire (Parker et al., 2013) by contrast has had research conducted into its psychometric properties, which has supported its validity and internal reliability. Both the UE‐ATR and the Experience of Therapy Questionnaire focus predominantly on negative therapeutic processes (McGlanaghy et al., 2021), and so are perhaps best used in conjunction with an additional measure that focuses on other potential negative experiences. The Negative Effects Questionnaire (NEQ; Rozental et al., 2016), for example, is a 32‐item measure, which focuses more on client experiences of negative effects.

### Negative effects in DBT

The focus within DBT research appears to have been on symptom reduction rather than on client or staff experiences. Research which is explicitly concerned with negative effects of DBT is even more rare. However, it should be noted that there is a wealth of grey literature detailing first‐hand client accounts of their negative experiences of DBT. For example, a blog post by Hollie Berrigan (2022) details the ways in which she found DBT to be rigid, poorly resourced, and not sufficiently trauma informed. Additionally, in 2021, Rebecca Donaldson started a Facebook group called ‘Stop Dialectical Behavioral Therapy’. The group now has over 800 members, some of whom gave permission to share their quotes in an article for Mad in America (Donaldson, 2022). One anonymous quote reads: ‘DBT was the worst thing that ever happened to me. I needed trauma therapy for years just to process the abuse that DBT was’.

In terms of formal research on client experiences of negative effects from DBT, there are two potentially relevant papers, which interviewed participants about their experiences of DBT (Hodgetts et al., 2007; McSherry et al., 2012). Although clients in these studies were not asked explicitly about negative effects, such experiences were raised. Indeed, the findings from these studies included participants experiencing DBT as overly structured, de‐humanising, inaccessible and lacking exploration of the past (Hodgetts et al., 2007; McSherry et al., 2012).

Another paper by Barnicot et al. (2022) involved participants being interviewed about their experiences with either DBT or mentalisation‐based therapy (MBT) and being given two self‐report quantitative measures to complete. Participants who had experienced DBT described difficult experiences with the therapeutic relationship, including therapists being hostile and critical. Finally, a report written by a collective of survivors of childhood sexual abuse and sexual violence, in response to the conceptualisation of and provision for those given a label of a personality disorder, made references to various negative experiences of DBT (Lomani, 2022). Indeed, the report describes how DBT services are structured around the concept of a ‘personality disorder’ and thus, despite some potentially helpful aspects of the intervention, many have experienced them as ‘pathologizing’ and ‘blaming’ (p. 7). The report goes on to discuss DBT as a behavioural approach, which some clients experienced as ‘silencing’ and ‘dismissive’ (p. 12), given that it is not trauma specific.

In terms of staff experiences of negative experiences from DBT, one paper by Kannan et al. (2021) interviewed 15 staff members who facilitated DBT in a college counselling centre and some relevant findings were identified. For example, there was an observation by clinicians that DBT did not adequately take into consideration cultural factors and that organisational constraints often interfered with the quality of service that was delivered (Kannan et al., 2021). One paper by Johnson and Thomson (2016) aimed to explore both staff and client experiences of DBT within the context of a forensic learning disability service. However, no interview questions were asked directly about negative effects, and perhaps in part as a result of this, only one negative experience was mentioned by both groups, namely that that DBT had been an intense experience.

Although the above research provides a useful starting point for understanding negative experiences of DBT, there was very limited exploration of what the impact of these experiences might be, or whether they may lead to any negative effects. Indeed, the paper by Barnicot et al. (2022) was the only identified piece of research to explicitly ask participants whether they had noticed any negative effects from DBT and to identify an increase in self‐harm and emotional distress as examples of this. However, the research by Barnicot et al. (2022) focused only on client experiences of DBT not staff, thereby potentially omitting key insights into the ways in which the two groups' understandings of negative effects compare.

### Rationale and aims

Given that DBT is recommended by the NICE (2009) guidelines and is thus widely practiced within the NHS, an understanding of its potential negative effects is important. The fact that this client group are at an increased risk of iatrogenic harm from psychological therapies (Fonagy & Bateman, 2006) further demonstrates the importance of this. However, no papers were identified which explicitly investigated both staff and client experiences of negative effects from DBT. This study aimed to address the gap in the literature and investigate potential negative effects from DBT, exploring both staff and client experiences and understandings of these effects.

## METHODS

### Participants

Eight participants who had received DBT and seven participants with experience of delivering DBT took part in the study. Participants were included if they were aged over 18, English speaking, and had experience of providing or receiving DBT within the last five years. Self‐reported demographic information for clients and staff can be found in Tables 1 and 2. In terms of service context, one staff participant had experience within an inpatient setting and six in community settings.

**TABLE 1:** Client participant demographics.

Name	Age	Ethnicity	Gender	Sexuality
Hallie	20–24	White British	Female	Asexual
Bea	20–24	White British	Female	Heterosexual
Harry	25–29	White British	Male	Other
Em	30–34	Black Grenadian and White British	Female	Heterosexual
Layla	30–34	White British	Female	N/A
Imogen	25–29	White British	Female	Bisexual
Amanda	30–34	White British	Female	Heterosexual
Sophie	25–29	White British	Female	Heterosexual

**TABLE 2:** Staff participant demographics.

Name	Age	Ethnicity	Gender	Sexuality
Benny	55–59	White British	Female	Lesbian
Nadia	35–39	White European	Female	Heterosexual
Paul	60–64	African Caribbean/British	Male	Heterosexual
Claire	35–39	White British	Female	Heterosexual
Ayah	30–34	Afghan – British	Female	Heterosexual
Oscar	40–44	White Irish	Male	Heterosexual
Eleni	30–34	White Other – Greek	Female	Heterosexual

### Procedure

Convenience and snowball sampling approaches were used for recruitment, via a combination of word of mouth and online advertisements. The majority of participants were recruited via online advertisements. To minimise the established risk within psychological research of the majority of research participants being White (Yancey et al., 2006), recruitment materials were also sent directly to organisations involved with the mental and emotional well‐being of racially minoritised adults. The sample size was determined by a combination of information power (Malterud et al., 2016) and pragmatism (Braun & Clarke, 2019), where information power is defined as a sample size large enough to meet the aim of the study, and pragmatism entails time and resources available.

A draft interview schedule for clients was partially informed by the Negative Effects Questionnaire (Rozental et al., 2016), and a draft interview schedule for staff was created drawing on information from the Unwanted Event to Adverse Treatment Reaction checklist (Linden, 2013). Both semi‐structured interview schedules sought to explore experiences of negative effects. Accordingly, they included initial questions about the overall experience of providing or receiving DBT followed by a series of questions that more specifically related to negative effects, including specific examples of negative effects, their understanding of negative effects, how long these negative effects lasted and how they were managed. Feedback from people with experience of receiving and providing DBT was sought before finalising the interview schedule. All interviews were conducted online and by the same person.

A simple transcription scheme based on conventions outlined by Banister et al. (2011) was used to guide the transcription process.

### Analytic approach

Thematic analysis is a method used to develop, analyse and interpret patterns within qualitative data (Braun & Clarke, 2022). By focusing on themes across the data set, thematic analysis facilitates an exploration of shared meanings, and as such was seen as an appropriate analysis technique for addressing the research questions of this study. Furthermore, thematic analysis has been indicated as appropriate for exploring participant's lived experiences, perspectives, and the factors that influence and shape certain phenomena (Braun & Clarke, 2013). In the present research, this was achieved by exploring staff and client lived experiences of the negative effects of DBT, and the factors that may have contributed to these.

This research used a reflexive thematic analysis approach, given that this is the most ‘fully qualitative’ and therefore most appropriate for exploring meanings and understandings (Braun & Clarke, 2022). On the spectrum of inductive to deductive reflexive thematic analysis, this research is situated more towards the inductive end, in recognition of the ethical importance of representing participant understandings (Swauger, 2011) and experiences.

Analysis was guided by Braun and Clarke's (2006) six‐phase process, which include familiarisation with the dataset, coding, generating initial themes, developing and reviewing themes, refining and naming themes, and writing up. The lead author adopted a manual coding process rather than using software, to facilitate deep engagement, as well as time for reflection and insight (Braun & Clarke, 2013).

### Ethics

University ethical approval was obtained. All participants were given a participant information sheet prior to engaging in the research, which informed them that all identifiable information would be removed from transcripts, and that all data would be held and processed in accordance with the Data Protection Act (2018) and General Data Protection Regulations.

At the beginning of each semi‐structured interview, it was reiterated that participants could take a break at any time, decline to answer questions or discontinue the interview. A process consent approach (Polit & Beck, 2010) was also adopted whereby consent is continuously re‐established in a collaborative way. At the end of each interview, time was allocated to check in with participants regarding their experiences of taking part in the study and any concerns that may have arisen.

Finally, each client participant was offered a £10 voucher for their time. It was made clear that the intention of providing vouchers was not to incentivise participation, rather to remunerate participants for their time, and not rely on free emotional labour (Faulkner & Thompson, 2021).

### Reflexivity

Researcher subjectivity and reflexivity is the key to conducting a successful reflective thematic analysis (Braun & Clarke, 2022). Given that reflexivity is never final, rather an ongoing process, the researcher kept a reflexive journal (Ortlipp, 2008) to reflect on each stage of the research process.

Given the lead author's experiences of both receiving DBT, and providing cover for a DBT facilitator, they occupied a complex mix of both ‘insider’ and ‘outsider’ researcher roles, which shifted depending on which dataset or participant group was being interacted with (Obasi, 2014; Paechter, 2013). Given the risk that self‐disclosure too early in an interaction can remove the focus from the participant (Dunlop et al., 2021), it was not felt appropriate to actively disclose these roles. However, as insider research can bring an increase in openness to research interviews (Keval, 2009; Watts, 2006), if directly asked about their inspiration for the research, the researcher named their lived experiences.

## RESULTS

**FIGURE 1:**
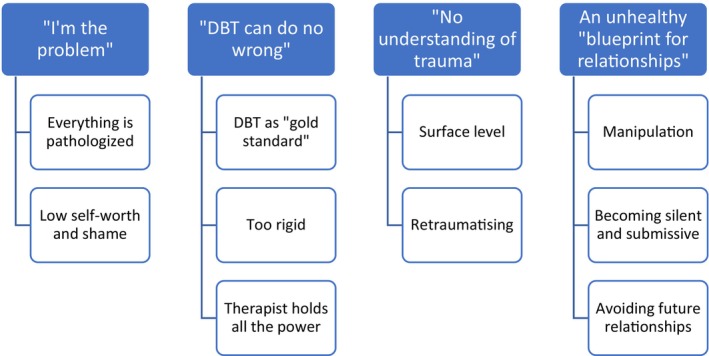
Client thematic map.

Four themes relating to client experiences of negative effects were generated (see Figure 1).

### Client theme one: ‘I'm the problem’

This theme relates to clients' experiences of having had ‘the problem’ placed within them, rather than within the systems around them or events they had experienced.

#### Everything is pathologised

Several participants reflected that their distress had been pathologised. In particular, the concept of ‘therapy interfering behaviour’ was discussed. Bea recalled that ‘my crying was a therapy interfering behaviour’ and Hallie summarised: ‘any resistance is categorized as a therapy interfering behaviour when actually any sane person would be resistant to this right now’.

Although one participant appreciated receiving a diagnosis because it meant that ‘it wasn't all in my head’ (Imogen), several others discussed the negative impact of having been given a label of ‘BPD’ and the role it played in pathologisation. Amanda explained that being labelled with ‘BPD’ meant that ‘if I had any problems with the therapy, it was very much just thrown in as a symptom’.

#### Low self‐worth and shame

Almost all participants described an experience whereby DBT had left them feeling ‘ashamed’ (Amanda) or had reduced their self‐esteem. Hallie shared, ‘a lot of the stuff that they do with the punishments makes you feel shame’ and Bea said, ‘I completely lost any self‐esteem and confidence I had because I thought I was a terrible person’. Some participants also reflected on the way in which they felt problematised, which led to feelings of shame. For example, Harry shared that ‘I was seen as a sort of troublemaker’ and Em recalled how the DBT team were ‘making it out like it was me … I thought there was something wrong with me’.

### Client theme two: ‘DBT can do no wrong’

The second theme captures the ways in which clients felt that DBT was marketed to them as an infallible protocol.

#### DBT as ‘gold standard’

Several participants mentioned that DBT was described as the ‘gold standard’ (Em) or ‘the only thing that's going to be effective’ (Layla). On occasion, this left clients with a sense that they should feel fortunate for having been offered DBT and could not report any concerns. As Sophie reported, ‘I didn't want to come across like I was just moaning when I'd waited so long to have this DBT’. The marketing of DBT as such an effective treatment also left some participants concerned that if they did not progress, there would be nothing else for them. As Hallie reflected, ‘where does that leave you? Does that mean you're un‐helpable?’

#### Too rigid

Several clients discussed the ways in which facilitators' beliefs that DBT is ‘always right, infallibly’ (Harry), led them to rigidly adhere to the manual as if it was a ‘Bible’ (Em) or ‘a script’ (Sophie).

Although one participant reported that ‘structure’ was helpful (Em), there was also an acknowledgment that the rigidity within DBT took this a step too far. The negative effect stemming from this was that clients felt as though their therapy was not person‐centred. When reflecting on the skills teaching, Sophie said, ‘it's not tailored to you’ and Hallie described how the ‘emphasis on adherence’ left facilitators without the ability to ‘take people's views into consideration’. Participants also discussed the ways in which the ‘rules‐based nature’ (Harry) of DBT put them under a great deal of pressure. As Harry said, the view of the facilitators was: ‘well it may not work for everyone, but you absolutely will fail if you don't follow every single rule’.

#### Therapists holds all the power

The belief that DBT is the ‘gold standard’ of therapy that facilitators should rigidly adhere to often led to a dynamic between therapist and client that Em described as ‘parent – child, or teacher – student’. Bea described feeling as though she had to ‘put my therapist on a pedestal’, and Hallie explained, ‘there's no scope for collaboration, and the therapist is always expert’. Several participants described their experience of DBT as being less like therapy and more like ‘school’ (Sophie). Indeed, Harry described his experience of DBT as a ‘very primary school‐like environment’ consisting of ‘constant control’. The impact of this was that clients often felt patronised and chastised, as illustrated by Amanda ‘I felt like I was this bad kid … the whole language and approach just feels very patronising, very punishment based’.

### Client theme three: ‘No understanding of trauma’

This theme references clients' reports that DBT staff did not appear to work in a trauma‐informed way.

#### Surface level

A common theme was participants feeling that DBT was not sufficient in addressing their trauma. Some participants even recalled being ‘forbidden’ (Harry) from talking about their past, apparently out of concern that they were ‘not emotionally strong enough’ (Em). Layla shared that her childhood trauma was put ‘in a box and *not* to be spoken about’, and Amanda explained, ‘to purely base everything on distraction when you're not dealing with the root of the problem … all you're doing is just sticking a plaster on and no wonder people are gonna [sic] bleed out’.

#### Retraumatising

Several participants conveyed that the DBT facilitators ‘had no understanding of trauma at all’ (Hallie), and that this led to several harmful practices, with the potential to retraumatise clients. Bea felt that ‘the whole process mimicked my childhood … it mimicked not being believed, it mimicked the invalidation’. Amanda, who at the time of engaging in DBT was living in an abusive home environment felt that the therapeutic ‘approaches and language felt the very same approach than what I was dealing with in the outside’. Sophie also found that the experience of support being withdrawn following self‐harm was potentially retraumatising. She recalled how being told to wait 24 hours to contact the team was experienced as ‘a rejection’ and ultimately ‘escalated’ her self‐injury.

### Client theme four: An unhealthy ‘blueprint for relationships’

The final client theme captures participants' experiences of the therapeutic relationship and the impact of DBT on other relationships in their lives.

#### Manipulation

Participants spoke both about feeling manipulated by DBT facilitators and feeling as though the interpersonal effectiveness module was teaching them how to be manipulative. Em felt as though she was being taught to manipulate people, which did not sit well with her because ‘it's not being honest and it's not being yourself’. Several participants reflected on the irony of this alongside the common misconception that people given a label of ‘BPD’ are manipulative. As Bea said, ‘you're telling me I'm manipulative and you're getting me to manipulate people’.

In terms of feeling manipulated by DBT, almost all participants touched on this in their interviews. Harry described DBT as ‘coercive’, and Bea stated that DBT staff ‘blackmailed me into so many situations’ when referring to the ways in which rewards were used to reinforce behaviour.

#### Becoming silent and submissive

Almost all participants mentioned that they had felt silenced by their DBT experience. Amanda shared how because of DBT ‘I was afraid to discuss concerns and I just became quite silent’, and Bea described DBT as ‘a very good way to silence people’. Several participants reflected on the way in which DBT had made them unsure of themselves. Em explained that a ‘culmination of things … made me not trust myself’, the impact of which was ‘I didn't stand up for myself as much’. Finally, Amanda described how she became ‘afraid to be assertive’. There was a sense that after having spent a length of time questioning their emotional responses in therapy and trying to please therapists, that clients then became ‘people pleasing and … compliant’ (Hallie) in other relationships.

#### Avoiding future relationships

Bea described how since engaging in DBT, ‘I didn't wanna [sic] make new friends. … I just became very isolated’. Hallie reflected that if the therapeutic relationship in DBT was the ‘blueprint for future relationships’, she ‘could never be in a relationship again’ and that she should never show her emotions because they could be ‘used against me’. Additionally, almost all participants described a negative impact on their relationship to help. Bea stated, ‘I completely disengaged from all services because I just couldn't trust them’, and Amanda reported that ‘I tend to … not call things like the duty lines. Now I don't speak out when I do feel bad’.

**FIGURE 2:**
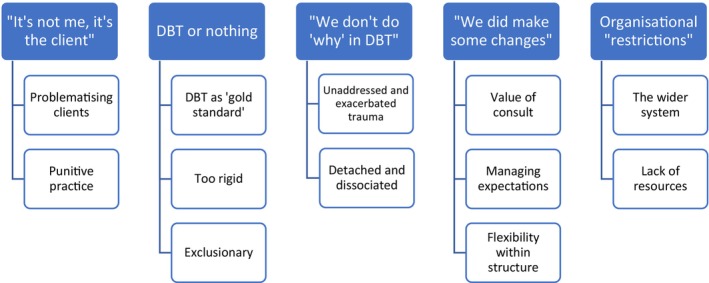
Staff thematic map.

Five themes relating to staff experiences of negative effects were generated (see Figure 2).

### Staff theme one: ‘It's not me, it's the client’

The first theme references instances in which staff saw members of their team placing ‘the problem’ within the client.

#### Problematising clients

Several staff participants raised concerns that DBT had the potential to place blame with the clients. Oscar outlined his worry that if clients were struggling to use DBT skills this could be framed as ‘on you’ rather than the responsibility of the DBT team.

Similar to the client sub‐theme regarding pathologisation, several staff members observed negative effects stemming from the label of ‘BPD’. Benny mentioned how one of her clients was ‘*furious* about being given *another* label’ and Claire noticed how the diagnosis could act as a ‘council of despair that professionals interpreted as unworkable with’.

#### Punitive practice

One of the ways in which clients were problematised was via ‘punitive ways’ (Oscar) of working. Whilst some of these punitive practices appeared to be related to the DBT protocol, in particular strategies such as ‘irreverence, extending, creating cognitive dissonance and creating some kind of dysregulation to teach skills’ (Ayah), others seemed linked to individual facilitators. As Ayah reported, ‘I've seen some of the interventions be used really, really effectively … and equally I've seen some of the interventions in certain hands be used in a not helpful way’. Staff also discussed the negative impact that punitive practice could have on clients, with Nadia explaining, ‘the narrative that then can develop out of that is: I'm really trying but … I'm just a bad client … somehow it must be my fault’.

### Staff theme two: DBT or nothing

This theme refers to the descriptions of DBT being viewed as a gold standard protocol to be upheld rigidly.

#### DBT as ‘gold standard’

Several staff described the ways in which DBT was viewed by teams as a ‘fix all’ (Ayah). Benny explained that DBT is often placed in a ‘white ivory tower’ and conceptualised as ‘the best thing’. The dangers of this were explored, with Nadia explaining, ‘to feel like the bad client in … a therapy that works for seemingly everybody else but for you is a really disheartening experience’.

Many participants also disclosed how DBT was often viewed by staff as a ‘last chance saloon’ (Benny), with the implication that if this therapy was not effective, there would be nothing else available.

#### Too rigid

All participants referenced the tendency for DBT to be ‘inherently a bit more rigid’ (Claire) than other therapeutic approaches and Oscar outlined his concern that DBT could be used in a ‘one size fits all’ way. Reflecting on the irony of this, Claire said, ‘for a therapy that is so much about, let's move from black and white into shades of grey, I think it struggles to hold shades of grey much of the time’.

Whilst it was noted that some clients found the structure helpful, it was equally acknowledged that for others this was not the case. Nadia noted that, ‘the very strictness … that for some of my clients has become a real positive, for others that's not been so easy’. When reflecting on the tendency for DBT to be practiced in a rigid way, Paul held the view that this was a misinterpretation of the therapeutic approach, and therefore a criticism not of DBT but of the facilitators. He explained: ‘Marsha Linehan acknowledged that that was never intended to be universal’.

#### Exclusionary

Staff outlined occasions on which DBT was not adapted for clients' needs and was therefore practiced in a way that could be viewed as exclusionary. One form that this took was ‘bombarding’ (Ayah) clients with skills. Paul also mentioned how inaccessible DBT terminology was for his clients and emphasised the importance of being able to ‘break it down and deliver it in a language that people can understand’.

Another form that exclusionary practice took was in failing to account for clients' social contexts. As Oscar summarised when reflecting on DBT skills, ‘they're quite socially constructed ideas of how one behaves … and what implicit assumptions are behind that? Class, power, culture, gender?’

### Staff theme three: ‘We don't do “why” in DBT’

The third theme references the many ways in which staff described DBT as not addressing trauma.

#### Unaddressed and exacerbated trauma

Many staff participants reflected on how DBT was more focused on the present than the past, and the implications of this on clients with trauma histories. As Eleni neatly summarised, ‘I think some people needed more work on trauma’. Oscar mentioned the importance of giving clients a ‘choice’ of DBT or ‘trauma related treatment’ and ‘transparency’ in describing DBT to ensure clients are aware that it is not primarily a trauma‐based intervention. Beyond the reports that DBT did not touch on trauma, there were also some participants who felt that it actively retraumatised clients. As Ayah explained, ‘actually it reinforced a lot of the messages that they would have had previously about … their emotions are too much’.

#### Detached and dissociated

Benny described concerns that DBT could feed into the ‘detached protector’ mode where clients become ‘slightly detached’ from their experiences. Oscar shared similar concerns, stating that DBT could exacerbate dissociation by encouraging ‘cognitive avoidance’.

When considering whether DBT could encourage avoidance, several participants mentioned how mindfulness may contribute to this. Indeed, Oscar stated, ‘some of them do dissociate and that's what makes mindfulness difficult’. Given these potential difficulties and the risk of mindfulness triggering dissociation, Paul emphasised the importance of having ‘an open discussion about mindfulness’ with all clients, in which the risks are outlined. However, from staff reports, it did not seem as though this happened often. Indeed, Eleni described how mindfulness exercises were often delivered as ‘a tick box task’.

### Staff theme four: ‘We did make some changes’

This theme entails the ways in which staff attempted to address some of the potential negative effects of DBT.

#### Value of consult

Almost all staff mentioned consult as a valued space in which any concerns about DBT could be discussed, and ideas for change could be generated. Eleni explained that ‘the consult helped … bring me back into the main principles but also gave me … the freedom… validated my tendency to go … into other interventions’.

Three participants discussed therapist‐interfering behaviours; a DBT concept, which states that just as clients can exhibit therapy interfering behaviours, so too can facilitators. As Ayah summarised, ‘one part of DBT is looking at therapist interfering behaviours as well. And again, if the consult is strong enough … it can be a helpful tool to manage some of that’.

#### Managing expectations

Four staff participants mentioned the importance of managing clients' expectations of DBT and not ‘putting it on a pedestal’ (Oscar). For example, Benny described how in pre‐treatment sessions she tells her clients, ‘It's just one more therapy. If it works, it works. If it doesn't, it doesn't’. Similarly, Nadia reported, ‘I try to make it really clear … where our limits are … what we can and can't do’.

#### Flexibility within structure

All staff mentioned ways in which they incorporated flexibility to ameliorate the tendency for DBT to be rigid. As Eleni summarised, ‘adherence – yes … but not getting … stuck into this idea and restricting yourself’. For example, Benny recalled how she had provided a client with an appointment time outside of regular hours, and Paul explained that he tends to use telephone coaching ‘not so rigidly’.

Finally, several participants described ways in which they had incorporated other therapeutic approaches into their practice, with Claire incorporating elements of cognitive analytic therapy, Eleni drawing on schema therapy, and Oscar working on ‘incorporating the body’.

### Staff theme five: Organisational ‘restrictions’

The final staff theme captures the ways in which the context surrounding the DBT team can contribute to negative effects.

#### The wider system

Several participants discussed the impact of the wider system that the DBT team sat within. Eleni mentioned that the wider system did not provide adequate DBT staff support, which left her wondering, ‘who contains the container?’ Oscar similarly touched on the concept of insufficient staff support from the wider organisation and the impact of this, saying, ‘good people get burnt out because they care and people who've stopped caring stay in their posts and I think that's the systemic risk’.

Oscar also described his concerns regarding the lack of training for DBT facilitators, saying, ‘you can do a really minimal training… and that can have an effect on overall care’.

#### Lack of resources

All staff participants mentioned a lack of resources within their DBT team. Benny explained ‘we don't have the funds to send out people the book’, and Nadia explained that she could not provide telephone coaching ‘simply because the Trust doesn't give me a telephone’.

Several participants also mentioned the impact that a lack of resources had on waiting times and amount of support available. Benny stated, ‘people were waiting up to two years for DBT’ and Paul expressed his frustration with the limited number of sessions per client that were funded.

## DISCUSSION

This research aimed to address the gap in the literature regarding client and staff experiences of negative effects from DBT. The first three themes generated from both sets of interviews can be broadly mapped onto one another and cover similar negative effects, including those stemming from the problematisation of clients, the rigidity of DBT and the lack of a trauma‐specific approach. In this way, staff and client understandings of negative effects can be viewed as similar. However, whilst several clients mentioned negative effects relating to the therapeutic relationship, this theme was not generated from staff interviews. In addition, staff participants regularly discussed organisational factors, which may have contributed to negative effects, and this was not a theme which was identified from the client transcripts. In these ways, client and staff understandings of negative effects differed.

The absence of clients mentioning wider organisational factors in relation to negative effects is perhaps understandable, given that when receiving DBT, they were likely not directly exposed to discussions around funding and service provision. Organisational factors that staff participants mentioned included high staff turnover rates, empathy burnout, insufficient staff training, long waiting lists, limited availability of sessions and inadequate physical resources. However, the fact that staff did not often discuss relationships is particularly interesting, given that difficulties within the therapeutic relationship are a key contributing factor to negative effects (Berk & Parker, 2009; Linden & Schermuly‐Haupt, 2014; Parry et al., 2016), and given that an entire module of DBT is dedicated to interpersonal effectiveness.

Consistent with the research by Barnicot et al. (2022), in which clients reported DBT therapists to be hostile and critical, participants in this research compared their interactions with DBT facilitators to abusive relationships. It is important to consider which aspects of DBT may contribute to this. One possible factor is the DBT technique of irreverence, which encourages therapists to behave in unexpected ways with the aim of shifting clients' thought processes. Examples of this include adapting a deadpan style of interaction or directly addressing sensitive topics (Linehan, 1993). Regarding negative effects resulting from these difficult therapeutic relationships, participants in this research described becoming less assertive, losing their sense of self, and also a more negative relationship to help.

Similarly to clients in the research by Hodgetts et al. (2007), McSherry et al. (2012) and Barnicot et al. (2022), participants in this study described the ways in which DBT was overly structured and rigid. Some participants found that due to strict adherence to protocol, they were unable to discuss the difficulties that they wanted to. Additionally, some participants explained that staff adhering strictly to a protocol resulted in a power imbalance whereby the therapist held all the knowledge and could never be wrong. This is an important finding, given that a power imbalance between therapist and client can result in negative effects (Berk & Parker, 2009; Linden & Schermuly‐Haupt, 2014; Parry et al., 2016). Perhaps in part due to the rigidity mentioned above, several participants found that there was no space to discuss trauma. Beyond being unable to talk about trauma, some participants in this research found DBT to be actively retraumatising, for example by mirroring abusive relational dynamics. This finding needs to be considered in relation to the research that has examined the safety and efficacy of DBT for clients with a trauma history (Zeifman et al., 2021) and it suggests potential avenues for future research.

In accordance with the client experiences of feeling blamed and pathologised, many staff participants observed team members locating ‘the problem’ within clients. One negative effect that staff observed to be originating from this problematisation was the use of punitive practice, whereby instead of working to understand what might underly a certain behaviour, clients were instead chastised for it. When invited to consider which aspects of DBT might contribute to these practices, one staff participant mentioned the technique of irreverence. This corresponds with research, which suggests that in some instances the use of irreverence can increase clients' distress (Swales & Heard, 2016). Furthermore, research has found that mental health practitioners have more negative views of clients given a diagnosis of ‘BPD’ than they do clients given other diagnostic labels (McKenzie et al., 2022).

In line with the client theme regarding the rigidity of DBT, several staff participants in this research referred to the inflexible nature of DBT and the negative effects resulting from this, such as power imbalances and exclusionary practice. These observations correspond with research by Curran et al. (2019), which outlined a causal relationship between therapist rigidity and clients feeling disempowered. A unique finding to this research was that rigidity was also present in the prescribing of DBT. Indeed, several staff participants described how DBT was framed as the only appropriate intervention, often leaving clients who did not find DBT helpful feeling hopeless.

Many staff in this research referred to the ways in which DBT was not a trauma‐specific approach. When reflecting on the negative effects stemming from this, staff described therapeutic relationships mirroring abusive histories, as well as clients becoming more detached from their emotions. Perhaps then, it is no surprise that the dropout rates for DBT are higher for those who have experienced childhood trauma (Euler et al., 2021).

With regards to how negative effects were addressed, all staff participants recognised the limitations of DBT and made valuable suggestions for amendments. These strategies broadly fell into three sub‐themes. The first sub‐theme described the value of consult in being able to address negative effects although, unfortunately, there is also evidence that approximately 10% of DBT teams do not use team consultation (Dubose et al., 2013). The second sub‐theme described the ways staff attempted to counteract the narrative that DBT was the gold standard of treatment. Finally, participants described some of the ways they attempted to introduce some flexibility into DBT.

### Clinical implications

Firstly, the findings from this research indicate the importance of employing methods to recognise negative effects. DBT clinicians should be asking themselves what harm looks like, what they might be doing that contributes to this, and what they can do to address any negative effects. In practice, however, despite all efforts to counteract this, it is likely that staff will regularly overlook certain negative effects (McGlanaghy et al., 2021). It is therefore of the utmost importance that clients have as many opportunities as possible to report experiences of negative effects. Methods to facilitate this could include regular reviews with therapists, in which clients are explicitly invited to reflect on any negative experiences (Curran et al., 2019), as well as opportunities for anonymous feedback. An additional organisational level response would be for teaching on negative effects to be included in the DBT accreditation training, as was recommended by Castonguay et al. (2010) to be the case for all therapies.

When considering both client and staff reports regarding the rigidity of DBT and resulting power imbalances, perhaps strict adherence to a protocol is not as important as was once thought. Indeed, guidelines now state that the NHS should provide care that is tailored to the personal preferences of service users (NICE, 2019 ). Perhaps then, DBT practitioners should, as suggested by one of the staff participants, use the DBT protocol as a guide rather than an absolute.

Given the findings regarding negative effects stemming from the therapeutic relationship, attention should be paid to the potential for this to cause harm. Irreverence, manipulation or any form of ‘withdrawal of warmth’ (Green, 2022) should not be used. In addition, particular attention should be paid to identifying and addressing the power imbalance inherent in the relationship, given its role in contributing to negative effects (Curran et al., 2019). Furthermore, given the regularity with which both clients and staff mentioned problematisation as a negative effect, it would be warranted for DBT services not to label clients as having a ‘personality disorder’, unless they explicitly request this (Lomani, 2022).

Finally, the findings from this study indicated that rigidity was also present in the prescribing of DBT, where perhaps an informed consent procedure would be more appropriate. Information based both on this research and previous research concerning negative effects of DBT should be made widely available to all, and especially to those being referred to DBT, who should then be able to choose if they feel that the intervention is suited to them. Indeed, the NHS guidelines on consent to treatment (NHS, 2022) state that for consent to treatment to be valid it must be voluntary and informed.

### Research implications

To the authors' knowledge, this study was the first to explicitly investigate staff experiences of negative effects of DBT. Staff participants described many ways in which DBT had resulted in negative effects, which raised the question of how these staff managed the knowledge that they had been part of teams that perpetrated harm. In particular, whether these participants encountered moral distress; an experience that arises when staff act in ways that they perceive to violate their professional integrity or are constrained from acting in ethically appropriate ways (Epstein & Hamric, 2009). Indeed, several staff participants in this study described ways in which organisational restrictions prevented them from practicing in the ways they would have wanted to. The ways that psychologists might respond to moral distress include support seeking, becoming silent and taking a stand against perceived injustices (Austin et al., 2010). Qualitative research to explore the ways that DBT facilitators respond to occasions on which they recognise the negative effects stemming from their practice would be of value.

Future quantitative research into this area would also be beneficial. In particular, a study with a larger and more representative sample of those who have experienced DBT, would be of value in providing an estimate of the prevalence of negative effects. The development of a negative effects from therapy questionnaire created in full partnership with experts by experience would be a key foundational aspect of such research.

Finally, whilst those who are marginalised are more likely to experience negative effects from therapy (Crawford et al., 2016), this research did not explore whether this was the case in DBT. However, given the findings in both previous research (Kannan et al., 2021) and in this study which indicated that DBT did not adequately consider cultural and social factors, further investigation into this is clearly warranted. In particular, future research into whether the negative effects of DBT are more likely to be experienced by those who are racially minoritised, members of the LGBTQIA+ community, disabled, or hold any other marginalised identity, would be of value.

### Strengths and limitations

This research succeeded in exploring both staff and client experiences and understandings of the negative effects of DBT, an area of research which until now has been largely unexplored. The use of a qualitative approach enabled the collection of rich data, which provided opportunities for previously untold stories (Pearce, 2007) and the lead author demonstrated commitment to the topic through ‘prolonged engagement’ (Yardley, 2000, p. 7), both as someone with lived experience of DBT, and through thorough and lengthy immersion with the data and literature. This was combined with time for distancing from the data, allowing space for reflection. This dual process of immersion and distance contributed to rigour in the processes of coding and theme generation (Braun & Clarke, 2022). Rigour was also demonstrated through the inclusion of direct participant quotations and the consultation work that informed the development of the interview schedules.

A potential limitation of this research is that it was not always clear whether the negative effects identified were specific to the DBT intervention, or whether they were in part related to therapist or organisational factors. Relatedly, there was limited information about the setting in which DBT was delivered and it was not possible to determine whether the DBT experienced by the client participants was adherent to the full DBT protocol on which the NICE guideline is based. It is therefore possible that some of the negative effects reported by client participants are attributable not to the DBT protocol itself, but to the ways in which it might be practiced. Indeed, there were indications from the staff participant responses that the DBT they had provided was not fully adherent to the protocol. For example, the references to the limited number of sessions due to resource issues and not being able to provide telephone coaching.

A strength of this research was the attempt to recruit participants who were racially minoritised, by making contact with relevant charities. However, despite this, the client participant sample was not representative of the UK population with regards to ethnicity, according to the latest census (Office for National Statistics, 2021). Also, in relation to recruitment, seven out of the eight client participants identified as female, mirroring an existing limitation within DBT research whereby those identifying as female are disproportionately represented (Wupperman & Edwards, 2017). In addition, there was no specific question or prompt regarding the social GGRRAAACCEEESSS (Burnham, 2013) included in the interview protocols, which may have resulted in contextual information being overlooked.

## CONCLUSION

It is likely that many of the negative effects identified in this research result from a combination of factors, including therapist factors, organisational factors, and poor fit (Parry et al., 2016). As such, it is also likely that other therapeutic interventions may cause similar negative effects. Indeed, clients who have experienced MBT have also found it to be too rigid (Barnicot et al., 2022), and clients who have experienced Cognitive Behavioural Therapy have also found it to be pathologising (Ratnayake, 2022). Nevertheless, the findings regarding staff observations of placing the problem within the client in DBT are the first of their kind and there are some features of DBT that may set it apart from other therapies with regards to negative effects. For example, the explicit suggestion for therapists to use ‘withdrawal of warmth’ as a therapeutic technique.

Based both on the findings from this research, and previous research, there are several adaptations that could be made to DBT to reduce the likelihood of negative effects occurring. Central to these changes would be the implementation of meaningful informed consent procedures so that no client is referred to DBT without being aware of the potential for both positive and negative effects.

## AUTHOR CONTRIBUTIONS

**Zazie Lawson:** Conceptualization; data curation; formal analysis; investigation; methodology; writing – original draft; writing – review and editing. **Lorna Farquharson:** Conceptualization; supervision; writing – review and editing.

## CONFLICT OF INTEREST STATEMENT

The authors declare no conflicts of interest.

## Data Availability

Due to the nature of this research, participants did not agree for all of the data to be shared publicly, but supporting quotes from the interviews are included in the article. Further details of the data collected and additional supporting quotes are available on request.
